# Multi-Objective Optimization of Thin-Walled Connectors in Injection Molding Process Based on Integrated Algorithms

**DOI:** 10.3390/ma18091991

**Published:** 2025-04-28

**Authors:** Size Peng, Mingbo Tan, Daohong Zhang, Maojun Li

**Affiliations:** 1 State Key Laboratory of Advanced Design and Manufacturing Technology for Vehicle, Hunan University, Changsha 410082, China; 2 Tianjin Key Laboratory of Aquatic Science and Technology, School of Environmental and Municipal Engineering, Tianjin Chengjian University, Tianjin 300384, China

**Keywords:** injection molding, multi-objective optimization, thin-walled structure, NSGA-II algorithm

## Abstract

For the manufacturing of thin-walled connectors, warpage represents an inherent challenge in injection molding, significantly affecting dimensional accuracy and shape consistency. This study introduces an optimization methodology that combines Latin Hypercube Sampling (LHS), numerical simulation, a DBO-BP neural network prediction model, and integrated multi-objective optimization algorithms (NSGA-II). Initially, LHS is employed to select experimental sample points, followed by numerical simulations to evaluate the influence of process parameters on the response variables. Based on the simulation outcomes and response data, a DBO-BP neural network prediction model is developed to enhance the precision of multi-objective optimization. Subsequently, the NSGA-II algorithm is utilized for multi-objective optimization to analyze the effects of various process parameter combinations on warpage, shrinkage, and clamping force, ultimately identifying the optimal Pareto front solutions. The optimization results demonstrate that the model’s prediction accuracy for warpage and volume shrinkage is within 5%. The clamping force remains relatively high, with the optimal values for warpage, volume shrinkage rate, and clamping force being 0.173 mm, 7.5%, and 15.83 tons, respectively. This approach facilitates the optimization of injection molding process parameters while ensuring the quality of thin-walled connectors, thereby improving production efficiency and minimizing defects.

## 1. Introduction

Plastic injection molding (PIM) is commonly utilized in industries such as automotive, home appliances, and electronics due to its advantages in efficient production, precise control, and the ability to manufacture complex shapes in large volumes. In the context of new energy vehicles, connectors, as critical components of the electrical system, face increasingly stringent performance requirements, such as heat resistance, reliability, and vibration resistance. At the same time, the structural diversity and fine precision requirements of connectors have led to a growing demand aimed at improving injection molding parameter settings [1]. During the injection molding process, there exists a complex geometric nonlinearity between process parameters, and different parameters may contradict each other, thereby affecting the molding quality. For example, increasing injection speed may help improve filling performance and reduce short shots, but it could also result in greater gas entrapment, leading to weld lines or surface defects. Lowering mold temperature might reduce warpage, but it may also cause uneven cooling of the material, resulting in uneven shrinkage or surface imperfections [2]. Systematic adjustment of process parameters is vital for reducing production costs and mitigating defects like warpage, volume shrinkage, weld lines, and incomplete filling in injection molding.

To gain deeper insights into how process parameters influence molding quality, researchers have conducted extensive studies. Mourya et al. [3] provided a detailed description of how variations in molding process parameters affect defects in plastic parts. Given the critical role that in-mold temperature control plays in improving warpage quality. Hopmann et al. [4] proposed a new strategy for initiating heating and cooling elements in advance to predict and regulate in-mold temperature. Chen et al. [5] optimized the velocity–pressure changeover point and the hold pressure according to changes in rod strain characteristics to ensure the stability of the injection-molded part’s quality. Gim et al. [6] utilized neural networks as surrogate models to study the impact of cavity pressure distribution on part quality. Chen et al. [7] discovered that temperature and melt temperature are key parameters in the optimization of PET bottle preform warpage.

With the development of computer-aided technologies, the integration of injection molding simulation with optimization design methods has become the mainstream approach for process parameter optimization, significantly reducing computation time and production trial costs. Moayyedian et al. [8] utilized methods such as Finite Element Analysis (FEA), the Taguchi method, and artificial neural networks (ANNs) to effectively identify optimal process parameters, achieving the best final product quality. Shen et al. [9] integrated artificial neural networks (ANNs) with genetic algorithms (GAs) to optimize the injection molding process. By incorporating the Taguchi design approach with Moldflow analysis, shrinkage-induced deformation during the injection process was effectively minimized, leading to improved roundness and concentricity of the component [10]. Mohammad et al. [11] employed the Taguchi method and numerical simulation to minimize sink marks and warpage in plastic gears. Feng et al. [12] applied a combination of the Taguchi design approach, ANOVA, and hybrid ANN-MOGA methods to automate the optimization process for thin-walled plastic products. Otieno et al. [13] minimized warpage and shrinkage through fuzzy logic and pattern search optimization methods. Lockner et al. [14] proposed a data-driven injection molding process parameter optimization approach using transfer learning based on inductive networks to decrease the quantity of data from the injection molding process required for neural network training. In the optimization of warpage and shrinkage, Otieno et al. [13] established a predictive model grounded in process parameters. The Taguchi L25 design was utilized to generate training datasets, and a fuzzy logic model was employed for defect forecasting. The model was subsequently refined using pattern search algorithms, which minimized the square root of the average squared error and error of the regression model, thereby enhancing the precision of the predictions. This methodology substantially augments both product quality and operational efficiency. Wu et al. [15] undertook an investigation into the nonlinear shrinkage of micro-small-module plastic gears, utilizing a response surface model and NSGA-II for multi-objective optimization. The results showed that the error rates for key shrinkage dimensions were all below 2%, with shrinkage variables reduced by 5.60%, 8.23%, 11.71%, and 11.39%, respectively. Additionally, the tooth profile deviations were reduced by up to 49.96%.

Moreover, the implementation of smart algorithms has gradually gained attention in injection molding process optimization. As a recent swarm intelligence approach, the Dung Beetle Optimization (DBO) algorithm exhibits remarkable performance in terms of convergence rate, solution precision, and stability [16]. Liu et al. [17] introduced a multi-strategy improvement in the DBO algorithm (MI-DBO) to enhance operational efficiency. Wang et al. [18] refined the standard dung beetle optimization algorithm by adding tent chaotic mapping for initial population generation, a golden sinusoidal approach for position updating, and the Lévy flight mechanism to achieve a balance between exploration and exploitation. Lu et al. [19] employed the improved method to optimize the parameters of the grinding process, focusing on balancing carbon emissions, processing time, and cost. The experimental results revealed that after optimization, carbon emissions, costs, and processing times were reduced by 11.7%, 7.7%, and 6.7%, respectively.

In addition to process parameter optimization, the optimization of mold structure and conformal cooling channels is also a crucial approach to improving injection molding quality and efficiency. Proper gate placement can effectively avoid filling defects and jetting issues. For parts with complex geometries that are difficult to parametrize, Walale et al. [20] proposed a method for optimizing the gate position on complex curved surfaces, significantly enhancing the optimization efficiency. Research has shown that adding rectangular inserts before the flow channel gate can optimize the melt heat distribution, improving the surface quality of injection-molded automotive components [21]. Compared to traditional straight-drilled cooling systems, conformal cooling systems offer more uniform and efficient cooling, thereby enhancing product quality and production efficiency. In the context of conformal cooling channel design, numerous optimization strategies have been proposed in the literature, including but not limited to helical configurations [22], linear (serpentine) arrangements [23], non-circular cross-sectional geometries [24], modular/parametric design approaches [25], and lattice/porous structures [26]. Furthermore, these primary cooling channel designs can be synergistically integrated with auxiliary techniques such as bubble generators or baffles [24], thereby achieving enhanced cooling performance through multi-physics optimization. Studies indicate that conformal cooling systems have a significant advantage in reducing weld line defects [27,28,29].

Despite the aforementioned research progress, there is a gap in existing studies on the injection molding of multi-porous thin-walled connectors, as well as on the complex nonlinear and conflicting relationships between multiple quality indicators, such as molding quality and production cost. Additionally, the high dimensionality and interdependence of process parameters further increase the difficulty of effective optimization. While simulation-based and data-driven methods show promising application prospects, their performance largely depends on the accuracy of the surrogate models and the efficiency of the optimization strategies used. The motivation for this study primarily includes the following aspects:

1. Multi-objective optimization is a critical issue in injection molding. The challenge is to optimize processes in a way that improves product quality, increases efficiency, and meets the growing demands for increasingly diverse injection-molded products. This requires identifying the extent to which process parameters influence the product.
2. Warping deformation is a significant problem in injection molding. To ensure dimensional accuracy and maintain product shape precision, it is necessary to minimize warpage, reduce shrinkage, and control clamping force.
3. This paper applies Dung Beetle Optimization (DBO) to improve the prediction accuracy of the BP neural network, thereby enhancing the performance of multi-objective optimization.
4. Typically, warpage, shrinkage, and clamping force cannot be simultaneously optimized to their ideal values. Therefore, trade-off analysis is conducted to make decisions regarding multi-objective optimization.

To achieve this, a methodology combining sampling strategies, numerical simulation, neural networks, and multi-objective optimization algorithms is proposed. Latin Hypercube Sampling (LHS) is used for sample point selection, followed by simulation analysis using Moldflow 2019 version to evaluate the results. With reference to the response points and experimental data, a predictive model is built using the DBO-BP neural network. Finally, NSGA-II is applied for multi-objective optimization to identify the solutions. Section 1 summarizes the current research directions and methods for optimizing injection molding parameters. Section 2 analyzes the multi-objective optimization problems in injection molding, including mathematical models, optimization objectives, design parameters, and trade-off analysis methods. Section 3 introduces the research methods and principles of the beetle optimization algorithm and NSGA-II used in this paper. Section 4 describes the multi-objective optimization analysis and verification of wire harness terminals as the research object.

## 2. Overview of Multi-Objective Optimization Problem

### 2.1. Multi-Objective Optimization Model

The injection molding process involves multiple key performance indicators and complex process constraints and requires a balance between production costs and efficiency. This process is inherently a multi-objective optimization problem, and its mathematical representation is given by the following core equations:

$$Find:x=\left[x_{1},x_{2},\ldots ,x_{n}\right]$$

$$Minimize\;F\left(x\right)=\left\{f_{1}\left(x\right),f_{2}\left(x\right),\ldots ,f_{k}\left(x\right)\right\}$$

$$Subject\;to:\;{x_{i}^{min}<x}_{i}<x_{i}^{max},\;i=1,2,\ldots ,n$$

$$f_{j}\left(x\right)>0,j=1,2,\ldots ,k$$

In this context, *x* represents the vector of process parameter combinations, where $x_{i}$(*i* = 1, 2, …, *n*) represents the *i*-th process parameter. Here, *n* represents the total number of process parameters. $f_{j}\left(x\right)$ (*j* = 1, 2, …, *k*) is the *j*-th objective function to be reduced, and *k* represents the total number of objective functions. $x_{i}^{min}$ and $x_{i}^{max}$ indicate the minimum and maximum values of the *i*-th process parameter.

### 2.2. Objective Functions

Warping is a shape deformation caused by uneven distribution of cooling-induced internal stresses through the injection molding procedure, and it is a member of the major issues affecting the characteristics of the molded products. It can lead to dimensional inaccuracies, appearance defects, and functional failures, ultimately affecting assembly performance and service life. Since warping directly impacts production efficiency and economic benefits, it is typically minimized as the first objective function, $f_{1}\left(x\right)$, in injection molding process optimization to enhance part quality and performance.

Volume shrinkage rate is an important quality indicator in injection molding, reflecting the change in the part’s volume during the cooling process. Excessive volume shrinkage can result in dimensional errors, warping, and appearance defects, which negatively impact product performance and assembly accuracy. Therefore, in process optimization, the volume shrinkage rate can act as the second goal function, $f_{2}\left(x\right)$, with the goal of minimizing it to optimize the dimensional precision and shape steadiness of the molded part while reducing the occurrence of defects.

Clamping force is a critical element in the injection molding process, as it keeps the mold closed during injection and prevents defects such as short shots or leakage. Insufficient clamping force can lead to incomplete filling, while excessive force can cause mold wear and increase production costs. Clamping force is selected as the third objective function, $f_{3}\left(x\right)$, in the course of optimization. The goal is to minimize clamping force while ensuring proper mold closure, improving production efficiency, reducing costs, and extending mold life without compromising part quality.

### 2.3. Design Variables

Table 1 enumerates the design parameters and their respective admissible ranges that constrain the optimization problem formulation. The process parameters—melt temperature ($T_{melt}$), mold temperature ($T_{mold}$), injection time ($t_{in}$), holding pressure ($P_{h}$), and holding time ($t_{h}$)—are considered as input variables. Melt temperature determines the flowability of the plastic melt. An appropriate melt temperature facilitates smooth filling of the mold, preventing issues such as incomplete filling and degradation. Mold temperature controls the cooling rate and the curing process of the molded part, ensuring uniform cooling and reducing warping and dimensional errors. Injection time directly impacts the completeness and density of the filling process. A reasonable injection time ensures rapid and uniform mold filling, preventing incomplete filling. Holding pressure is applied after injection to compensate for voids created by shrinkage, and an appropriate holding pressure can reduce warping and dimensional deviations while improving part density. Holding time determines the duration of the holding pressure, and adequate holding time ensures complete filling of the mold and prevents excessive shrinkage during cooling. By optimizing these parameters, the quality and accuracy of the injection-molded parts can be effectively improved.

### 2.4. Trade-Off Analysis

The purpose of multi-objective optimization problems is typically to minimize all objectives simultaneously. However, due to potential conflicts or the non-comparability among different objectives, identifying a single solution that simultaneously optimizes all objectives is often not possible. As a result, such problems do not produce a single optimal solution but instead generate a set of Pareto optimal solutions, each representing a trade-off between the competing objectives. This article explores the distribution of Pareto front solutions through the NSGAII optimization algorithm, considering that warping and shrinkage are equally important and greater than the locking force. Therefore, the multi-objective weights in this article are determined manually.

## 3. Optimization Methodologies

### 3.1. Multi-Objective Optimization Framework

**Figure 1:**
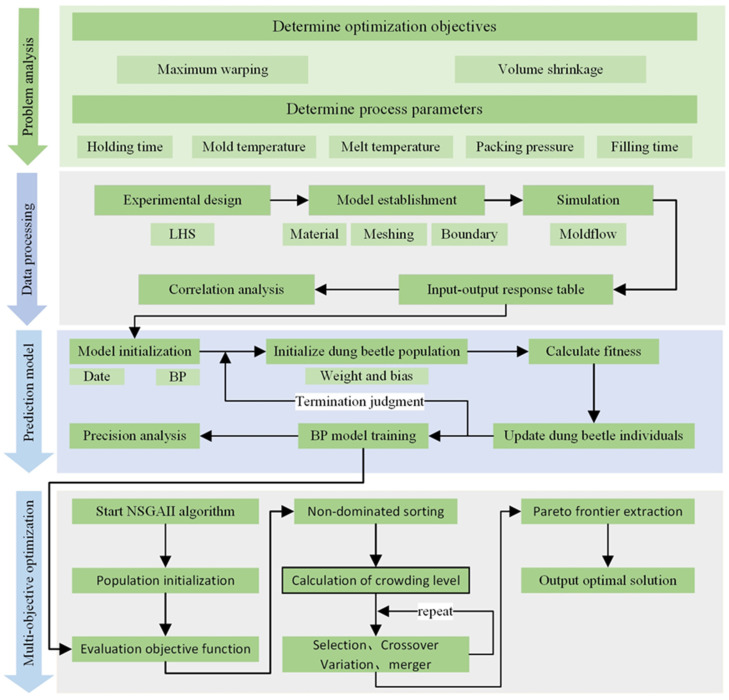
Flowchart of the proposed method.

A process parameter optimization method based on sampling strategies, numerical simulation, surrogate modeling, and multi-objective optimization algorithms has been developed to optimize the PIM process parameters with the objective of reducing warpage and volumetric shrinkage. The design space is discretized using a structured sampling approach, generating point distributions at multiple parameter level configurations. Concerning each sample point, numerical simulations are performed using Moldflow to compute the responses (warpage, volumetric shrinkage, and clamping force). In light of the sample data points and their associated responses, surrogate modeling techniques are used to approximate the mathematical relationships between design variables (process parameters) and responses, constructing a BP neural network model to compute the system responses corresponding to arbitrary sampling locations within the global design space. Subsequently, Pareto-optimal solutions are obtained through NSGA-II implementation, with the metamodel predictions constituting the multi-objective fitness evaluation. The architecture of the multi-objective process parameter optimization system for PIM is visualized in the flowchart presented in Figure 1. The principal stages of this approach encompass the generation and analysis of sample points, the development of response prediction models, and the execution of multi-objective optimization. The comprehensive procedure can be outlined as follows:

Step 1: The system responses are designated as optimization objectives, while the corresponding process parameters are identified as design variables, with their respective operating ranges specified as boundary constraints within the optimization framework.

Step 2: The Latin Hypercube Sampling (LHS) technique is employed to generate a space-filling, statistically representative set of sample points across the multidimensional design space. These sampled configurations are subsequently evaluated via high-fidelity numerical simulations in Moldflow to quantify their corresponding response metrics. In parallel, a global sensitivity analysis is conducted to identify and rank the dominant design variables exerting the most significant influence on the specified performance objectives.

Step 3: Develop a BP neural network model utilizing surrogate modeling techniques. Employ the sample points gathered in Step 2 as the training dataset and construct an enhanced DBOBP neural network model, evaluating its performance through the cumulative error.

Step 4: The multi-objective optimization framework utilizes NSGA-II to explore the parameter space and determine the Pareto frontier of optimal process conditions. Establish the initial parameters for the NSGA-II framework, and subsequently employ the predicted outcomes from the network model developed in Step 4 as the objective function for global multi-objective optimization to identify the Pareto optimal set. A subsequent trade-off analysis is conducted on the Pareto optimal solutions to determine the optimal compromise solution.

### 3.2. DBOBP-Optimized Neural Network Model

**Figure 2:**
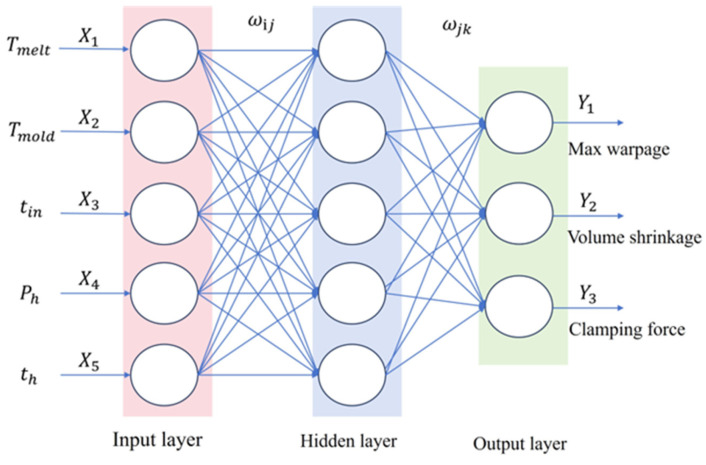
Neural network training diagram.

BP Neural Network (Backpropagation Neural Network) is extensively applied across disciplines, including classification, regression, and pattern recognition, due to its strong nonlinear fitting ability and adaptive learning characteristics. The network architecture shown in Figure 2 incorporates the following three sequential layers: an input layer for data acquisition and scaling, a hidden layer with sigmoid activation functions for pattern recognition, and an output layer that delivers the system’s predictions. This structure facilitates the modeling of complex input-output relationships in engineered systems. However, BP networks have some limitations, such as the tendency to get trapped in local optima, high computational cost, and overfitting issues, which affect their performance in handling complex tasks. To overcome these challenges, the Dung Beetle Optimization (DBO) algorithm has been suggested for the optimization of the weights associated with BP networks. DBO effectively avoids local optima through a global search mechanism, thus improving the network’s accuracy and generalization ability and enhancing its performance in complex tasks. The calculation principle of dung beetle optimization (DBO) is as follows [16]:

The Dung Beetle Optimization (DBO) algorithm is inspired by five typical behaviors of dung beetles—rolling, dancing, breeding, foraging, and stealing—each representing a unique search strategy. The rolling behavior, for example, simulates the beetle’s straight-line navigation relying on celestial markers (e.g., the Sun and Moon) while pushing dung balls. This enables adaptive positioning in a dynamic objective space and can be mathematically formulated as follows:

(1) $$x_{i}\left(t+1\right)=x_{i}\left(t\right)+k\times \sigma \times x_{i}\left(t-1\right)+b\times ∆x$$

(2) $$∆x=\left|x_{i}\left(t\right)-X^{w}\right|$$

In the equation, *t* signifies the present iteration and $x_{i}\left(t\right)$ denotes the spatial location of the *i*-th dung beetle at the *t*-th iteration. The parameter k∈(0,0.2] is a fixed constant that represents the deviation coefficient, and b∈(0,1] is a random number. σ=±1, where 1 indicates no deviation and −1 represents a deviation from the original direction. In this study, to simulate the complex environment of the real world, a probabilistic approach is used to set σ to either 1 or −1. xxx represents the environmental change, and *X* represents the worst position in the existing population.

Upon encountering an obstruction that prevents further movement, the dung beetle adjusts its trajectory by “dancing”, thereby reorienting itself to establish an alternative route. The positional adjustment at this stage is governed by a tangent function, as represented by the following equation:

(3) $$x_{i}\left(t+1\right)=x_{i}\left(t\right)+\tan\left(\theta \right)\left|x_{i}\left(t\right)-x_{i}\left(t-1\right)\right|$$

In the equation, $\tan\left(\theta \right)$ denotes the deviation angle. Due to the periodic nature of the tangent function, only the values residing in the interval [0, π] are relevant. Term $\left|x_{i}\left(t\right)-x_{i}\left(t-1\right)\right|$ represents the displacement of the i-th dung beetle between the *t*-th and (*t* − 1)-th iterations. It is essential to note that for *θ* = 0/π, 2/π, tan(*θ*) is undefined, resulting in no change in the beetle’s position.

The boundary delineation strategy is mathematically formulated as follows:

(4) $$\left\{\begin{array}{c} {Lb}^{*}=\max\left(X^{*}\times \left(1-R\right),Lb\right)\\ {Ub}^{*}=\min\left(X^{*}\times \left(1-R\right),Ub\right) \end{array}\right.$$

(5) $$R=1-\frac{t}{T_{max}}$$

Within the update rules, X* maintains the current best-known position, dynamically updated through pairwise comparisons across the population’s objective vectors. *Lb* refers to the lower limit of the search domain, while *Ub* represents the upper boundary. ${Lb}^{*}$ and ${Ub}^{*}$ define the lower and upper limits of the egg-laying zone, respectively. *R* indicates the inertia coefficient, and $T_{max}$ represents the maximum iteration count.

Upon identifying a secure location, the female dung beetle chooses this area to deposit its reproductive sphere. The velocity-driven relocation of the breeding particle in the solution space is mathematically described by the following equation:

(6) $$B_{i}\left(t+1\right)=X^{*}+b_{1}\times \left(B_{i}\left(t\right)-Lb^{*}\right)-b_{2}\times \left(B_{i}\left(t\right)-Ub^{*}\right)$$

$B_{i}\left(t\right)$ denotes the position of the *i*-th dung beetle at the *t*-th iteration, while $\;b_{1}\;$ and ${\;b}_{2}\;$ are two independent random vectors of size 1 × D, where D is the dimensionality of the optimization problem. The position of the breeding sphere is strictly confined within a certain range, known as the oviposition area. This foraging region also employs a dynamic boundary approach:

(7) $$\left\{\begin{array}{c} {Lb}^{b}=\max\left(X^{b}\times \left(1-R\right),Lb\right)\\ {Ub}^{b}=\min\left(X^{b}\times \left(1-R\right),Ub\right) \end{array}\right.$$

$X^{b}$ identifies the universal optimum configuration, while the specification of the ideal foraging zone boundaries [${Lb}^{b}$, ${Ub}^{b}$] enables the derivation of dung beetle foraging dynamics through the following position update operator:

(8) $$x_{i}\left(t+1\right)=x_{i}\left(t\right)+C_{1}\times \left(x_{i}\left(t\right)-Lb^{*}\right)-C_{2}\times \left(x_{i}\left(t\right)-Ub^{*}\right)$$

The decision variable $x_{i}\left(t\right)\;$ encodes the *i*-th optimizer’s candidate solution at generation t in the n-dimensional search space. The parameter $C_{1}$ follows a standard normal distribution N(0,1), whereas $C_{2}$ is uniformly sampled from the unit interval U(0,1). The resultant displacement dynamics for the thieving dung beetle follow this stochastic update rule as follows:

(9) $$x_{i}\left(t+1\right)=X^{b}+S\times g\times (\left|x_{i}\left(t\right)-X^{*}\right|+x_{i}\left(t\right)-X^{b})$$

Within the update rule, the decision variable $x_{i}\left(t\right)$ represents the current position of the *i*-th resource-acquiring optimizer at generation *t*. *g* is a stochastic vector of dimension 1 × D, drawn from a normal distribution, and F is a fixed constant.

### 3.3. NSGA-II Is Used to Locate the Pareto Optimal Solution

**Figure 3:**
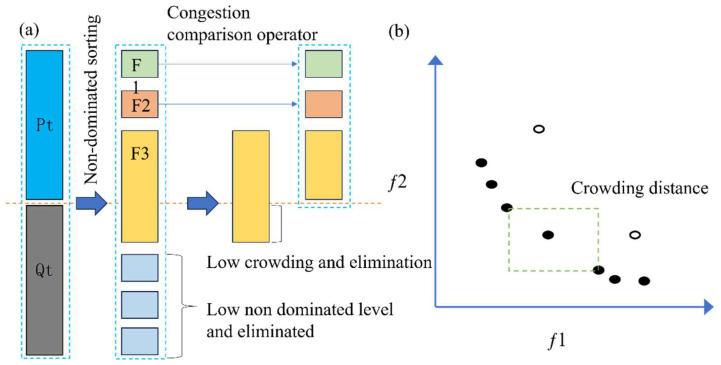
The key to NSGAII: (**a**) elite-preserving operator and (**b**) crowding distance.

As shown in Figure 3, NSGA-II is a robust genetic algorithm for multi-objective optimization, extensively applied to address intricate optimization challenges involving competing objectives. Its core principles include non-dominated sorting, congestion comparison, and elite strategy. It can layer the solution set through non-dominated sorting to ensure that excellent solutions are retained first and maintain the diversity of solutions through congestion calculation. NSGA-II preserves the optimal solution of parents and children through elite strategy so as to promote the algorithm to converge to the Pareto frontier quickly. Its main advantage is that it can optimize multiple objectives at the same time and effectively balance the convergence and diversity. It can provide a set of optimal Pareto solutions for decision makers to help make more comprehensive and scientific decisions.

### 3.4. Data Analysis

#### 3.4.1. Sensitivity Analysis

Sensitivity analysis is utilized to determine the critical process parameters (design variables) that significantly influence the response (objectives). This approach aids in eliminating irrelevant parameters, thereby reducing the dimensionality of the design space. The sensitivity between the process parameters and the response is quantified using the correlation coefficient, which can be computed employing the subsequent equation:

(10) $$\rho =\frac{Cov\left(X,Y\right)}{\sqrt{Var\left(X\right)Var\left(Y\right)}}=\frac{E\left[\left(X-\mu x\right)\left(Y-\mu y\right)\right]}{\sigma x\sigma y}$$

The statistical relationship between random vectors *X* and *Y* is characterized by the $Cov\left(X,Y\right)$ measures joint variation, *Var*(*X*) and *Var*(*Y*) quantify individual spread, and *ρ* ∈ [−1, 1] indicates linear dependence.

#### 3.4.2. Prediction Accuracy Analysis

For the concurrent engineering optimization tasks, the response model is utilized as the objective function in the optimization process for the NSGA-II algorithm. The predictive capability of the metamodel critically affects the quality of obtained Pareto solutions in engineering design optimization, potentially leading to non-convergence of the optimization algorithm. Hence, the model’s predictive performance is evaluated through three key metrics: root mean square error (RMSE), quantifying average deviation magnitude; mean absolute error (MAE), measuring absolute discrepancies; and coefficient of determination (*R*^2^), assessing explained variance, each computed by comparing simulated responses with model predictions.

(11) $${RMSE}_{j}=\sqrt{\frac{1}{n}\sum_{k=1}^{n}{\left(y_{j}-{\hat{y}}_{j}\right)}^{2}}\;\;j=1,2,3,\ldots ,k$$

(12) $${MAE}_{j}=\frac{1}{n}\sum_{k=1}^{n}\left|y_{j}-{\hat{y}}_{j}\right|\;\;j=1,2,3,\ldots ,k$$

(13) $$R_{j}^{2}=1-\frac{\frac{1}{n}\sum_{k=1}^{n}{\left(y_{j}-{\hat{y}}_{j}\right)}^{2}}{\frac{1}{n}\sum_{k=1}^{n}{\left(y_{j}-\bar{y}\right)}^{2}}\;j=1,2,3,\ldots ,k$$

*k* is the total number of objectives; *n* is the number of validation sampling points. Here, $y_{j}$ and ${\hat{y}}_{j}$ represent the *j*-th response for the *k*-th sampling point predicted by the respective response predictor and obtained from simulation experiments, respectively. $\bar{y}$ represents the mean of the *j*-th response.

## 4. Case Study

### 4.1. Finite Element Simulation Model

**Figure 4:**
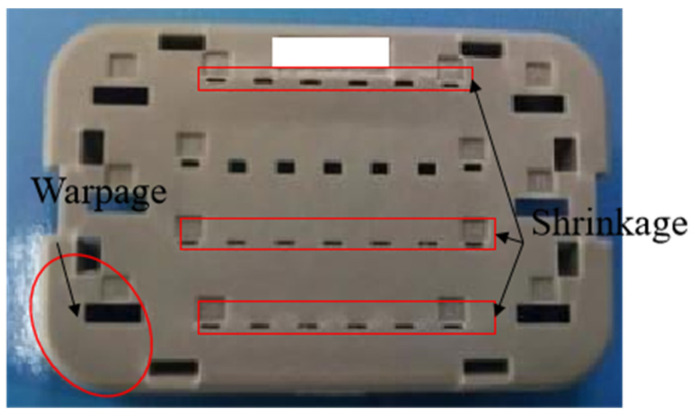
Details of injection-molded parts.

The injection-molded component’s three-dimensional structural model measures 31.60 mm × 20.08 mm × 8.20 mm, with a thickness of 1.0 mm. The design of this component is highly intricate, incorporating several holes, rib structures, and detailed surface features. The part drawing is shown in Figure 4. There is serious size shrinkage in the middle hole and obvious warpage deformation in the corner area.

**Figure 5:**
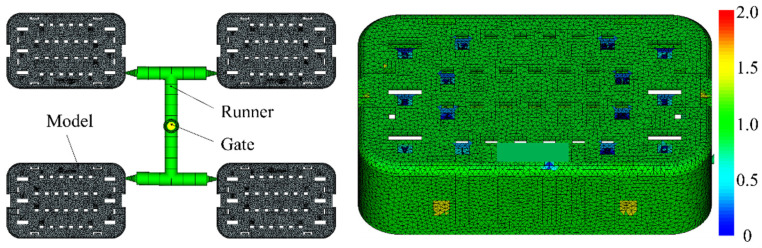
Mesh division model diagram and mesh division model diagram.

The finite element representation of the polymer injection process is mathematically formulated through Figure 5, designed to meet the manufacturing requirements of a four-cavity mold. The type and quality of mesh discretization significantly affect the numerical simulation results; therefore, selecting the appropriate mesh type and size is crucial. In this study, the terminal model for the automotive wire harness is a small, thin-walled component. A triangular element mesh is employed, with thickness diagnosis shown in the figure. The overall model thickness is 1 mm, with a greatest thickness of 2 mm. The mesh parameters for the triangular elements are outlined in Table 2. The peak aspect ratio of the mesh is 19.13, as illustrated in Figure 5, guaranteeing alignment with Moldflow’s dual-layer mesh analysis, with a geometric conformity standard surpassing 90%.

**Figure 6:**
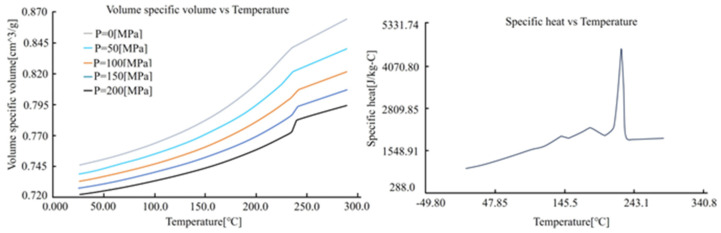
Temperature dependence of volume, specific volume, and specific heat.

The material selected for the wire harness terminal is PA66GF25, and the material property curve is shown in Figure 6. This material offers high strength, high-temperature resistance, excellent electrical insulation, chemical corrosion resistance, and wear resistance. These characteristics make it ideal for manufacturing electric vehicle wire harness terminals, as it can withstand high mechanical stress and temperature environments, ensuring electrical safety and long-term stability. The minimum and maximum limits of the design variables are presented in Table 3, determined based on recommendations from Moldflow and the manufacturer. Finally, the Latin Hypercube sampling method is employed to establish the experimental analysis table, and after conducting Moldflow simulations, the results are entered into the table.

### 4.2. Finite Element Simulation Analysis

**Figure 7:**
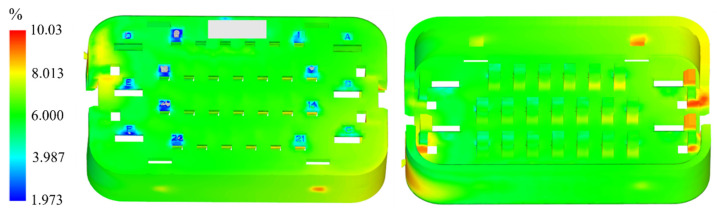
Simulation of volume shrinkage rate variation.

After configuring the material properties, mesh, boundary conditions, and process parameters, Moldflow was utilized for injection molding simulation analysis. The simulation results are presented in Figure 7. The overall volume shrinkage rate of the model is approximately 6%, with a maximum of 10% occurring at the filling end and areas with increased wall thickness. This behavior is attributed to the volume contraction effect of the molten plastic during the cooling phase and solidification. In regions with thicker walls, the cooling rate is slower, leading to higher shrinkage in these areas. The blue region in the figure is distributed at the positions indicated by the numbers. This is due to the small size at the marked positions and the smaller cross-sectional area of the local flow channels, which causes resistance to the flow of the melt. As a result, heat dissipates more quickly in these localized areas, causing a rapid decrease in the temperature of the melt front and leading to its quick cooling and solidification. Therefore, the phenomenon of the melt cooling and solidifying at the moment the cavity is filled occurs.

**Figure 8:**
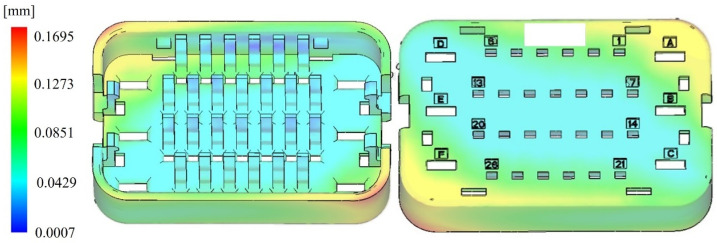
Simulation of warpage variation.

**Figure 9:**
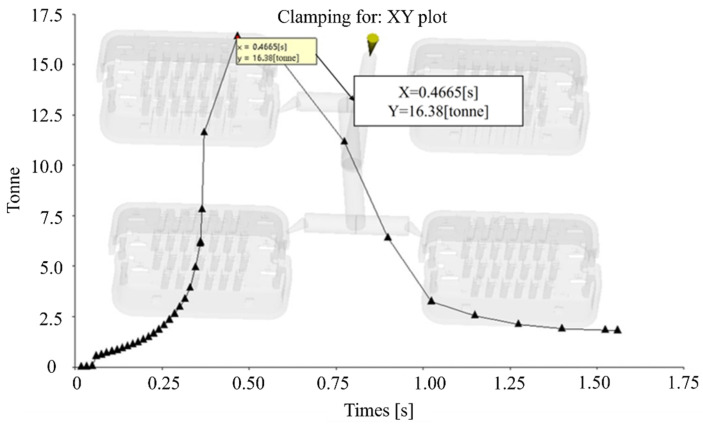
Simulation of clamping force.

The maximum warpage occurs at the lower-left and upper-right corners, as illustrated in Figure 8. The warpage deformation at the center of the model is minimal, with an average warpage of 0.085 mm and a maximum deformation of 0.170 mm. This can be explained by the gate location near the upper-right corner, where molten plastic initially flows into the mold and cools rapidly. Consequently, areas near the gate experience faster cooling, while regions farther from the gate, such as the lower-left and upper-right corners, cool more slowly. This uneven cooling results in greater shrinkage in the areas distant from the gate, generating significant internal stresses that lead to warpage deformation. The simulation outcomes of the clamping force are presented in Figure 9. Due to the small size of the wire harness terminal, the clamping force is relatively low, with a maximum value of 16.38 tons at 0.4 s. The trend of the curve shows that the clamping force is maximum when the melt fills the mold cavity and gradually decreases as the holding pressure time progresses. This is because, during the melt filling phase, the mold needs to withstand a large pressure to ensure the cavity is fully filled and impede the melt from retracting. In the holding pressure stage, the cavity is almost completely occupied by the melt, and the main purpose is to compensate for the shrinkage of the melt and maintain the filling pressure, so the clamping force gradually decreases until the holding pressure stage is completed.

### 4.3. Numerical Results Analysis

#### 4.3.1. Sensitivity Analysis

A total of 40 sample points were generated using the Latin Hypercube Sampling (LHS) method with stratification. For each sample, process parameters were set accordingly and evaluated sequentially using Moldflow simulations. The corresponding maximum warpage and volumetric shrinkage were obtained for each sampling point as the output responses. The correlation coefficient is calculated according to the obtained input and output response table. The correlation coefficient represents the sensitivity between the design variable and the response, and the value within each sub-box illustrates the correlation coefficient between two paired variables. A positive value denotes a direct correlation, a negative value signifies an inverse correlation, and a zero value suggests the absence of a relationship. The higher the absolute magnitude of the correlation coefficient, the more pronounced the association between the two variables.

**Figure 10:**
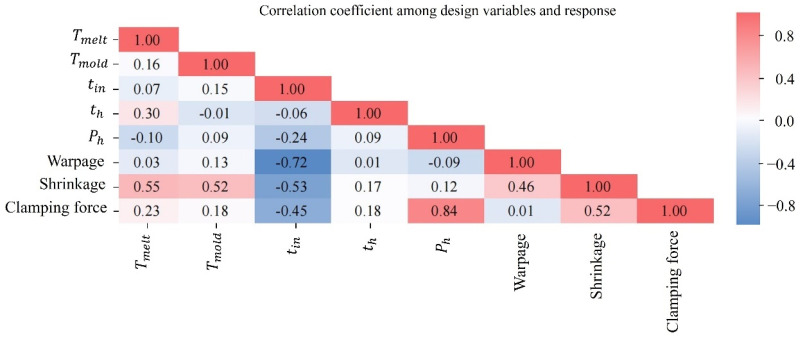
Relationship between design variables and response.

Based on the heatmap in Figure 10, it is evident that the injection time and clamping pressure have a significant negative impact on warpage, with correlation coefficients of −0.72 and −0.09, respectively. This indicates a strong negative correlation between filling time and warpage, while the effect of holding pressure on warpage is relatively minor. Mold temperature has a positive effect on warpage, with a correlation coefficient of 0.13. Similarly, filling time negatively influences shrinkage, with a correlation coefficient of −0.53, while the molten material temperature and mold thermal conditions exhibit positive correlations with shrinkage, with correlation coefficients of 0.55 and 0.52, respectively. Filling time also negatively affects clamping force, with a correlation coefficient of −0.45. Holding pressure, melt temperature, mold temperature, and holding time all positively influence clamping force, with correlation coefficients of 0.84, 0.23, 0.18, and 0.18, respectively. Based on the analysis, filling time is identified as a primary influencing factor because it has a significant negative effect on warpage, shrinkage, and clamping force. The correlation coefficients of filling time with warpage (−0.72), shrinkage (−0.53), and clamping force (−0.45) indicate that changes in filling time have a noticeable effect on these target qualities. A longer filling time tends to result in lower warpage and shrinkage, but it may lead to a decrease in clamping force as the melt pressure reduces over time. The strong correlations between filling time and these factors highlight its fundamental significance in controlling the injection molding process and product quality. Filling time is a key parameter in the optimization process, directly affecting dimensional accuracy, surface quality, and overall molding performance. While reducing filling time can improve efficiency, it may also increase warpage and shrinkage. Therefore, balancing filling time is crucial for optimal molding quality.

#### 4.3.2. Accuracy Analysis for Response Prediction

**Figure 11:**
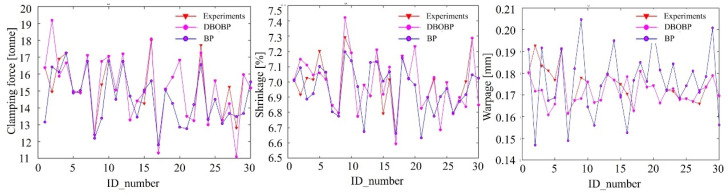
Accuracy prediction of DBOBP.

Using the Latin Hypercube Sampling (LHS) method, 40 initial sampling points were selected, with 30 data sets utilized for model training and 10 data sets employed to assess the predictive accuracy of the model. A prediction model was constructed based on DBO-BP, with a population size of 50, a maximum number of generations set to 100, and a *p* value of 0.3. The model training process and prediction results are shown in Figure 11. The results clearly demonstrate that the BP model optimized by DBO performs significantly better than the traditional BP model, demonstrating superior training effectiveness.

To quantitatively assess the performance of the prediction model, metrics such as MSE, RMSE, MAE, MAPE, and $R^{2}$ were applied. These performance metrics are calculated and presented in Table 4. Smaller values of MAE and RMSE indicate higher prediction accuracy, while an $R^{2}$ value closer to 1 reflects better model precision. Additionally, smaller MAPE values indicate lower relative error and greater precision of the model.

#### 4.3.3. Multi-Objective Optimization Results

**Figure 12:**
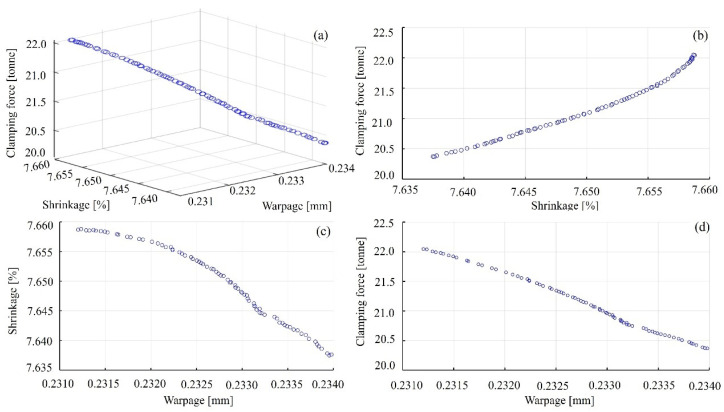
Pareto frontier of three-objective optimization result for the variations of (**a**) clamping force, shrinkage and warpage, (**b**) clamping force and shrinkage, (**c**) shrinkage and warpage, (**d**) clamping force and warpage.

The trained DBOBP model was used as the objective function, and the NSGA-II algorithm was applied for multi-objective optimization. In the NSGA-II algorithm for multi-objective optimization, the population size was set to 200, with a maximum of 300 iterations to ensure that the algorithm could thoroughly explore the design space and obtain a solution set as near as attainable to the Pareto optimal frontier. The Pareto front of warpage, shrinkage, and clamping force is illustrated in Figure 12. As indicated by the three-objective Pareto front, the Pareto points are approximately aligned along a straight line, suggesting the existence of a set of process parameters that can simultaneously achieve the optimal values for some of the objectives. However, it is infeasible to concurrently optimize all three objectives, as an optimal solution to the problem cannot be attained, necessitating a trade-off solution.

Figure 12b presents the Pareto front, showing the trade-off between warpage and shrinkage. Based on the correlation coefficient of 0.46 between warpage and shrinkage (Figure 10), they exhibit a positive correlation and fluctuate in a similar direction. Melt temperature, mold temperature, filling time, and holding time all exert a similar influence on both warpage and shrinkage. Nonetheless, the impacts of holding pressure differ as follows: a lower holding pressure results in a smaller shrinkage rate but leads to greater warpage. Additionally, warpage is strongly influenced by filling time, while shrinkage is strongly influenced by filling time, melt temperature, and mold temperature.

Figure 12c depicts the relationship between warpage and clamping force, highlighting the inherent trade-off between these two factors. The distribution of the Pareto points indicates that these two objectives exhibit a very similar variation trend. When optimizing one objective, the other is simultaneously improved, and both can reach their optimal values. As shown in Figure 10, the correlation coefficient between warpage and clamping force is weak (0.01), indicating a positive correlation with similar directional changes. Meanwhile, holding pressure has a significant impact on clamping force, with a correlation coefficient of 0.84. Similarly, Figure 12d shows the trade-off between shrinkage and clamping force. The distribution of the Pareto points suggests that reducing shrinkage simultaneously leads to a decrease in clamping force, allowing both objectives to reach their optimal values.

To visualize the trade-off relationships among the objectives, we presented 2D projections of the Pareto front. In some of these projections, the distribution of points appears nearly linear, which is mainly due to the strong correlations between certain objectives. For example, clamping force and shrinkage exhibit a strong positive correlation, meaning they tend to increase or decrease together as process parameters change. Conversely, warpage shows a negative correlation with both shrinkage and clamping force, indicating that increased shrinkage or clamping force often leads to reduced warpage. These strong linear or nonlinear interdependencies cause the Pareto-optimal solutions to cluster along certain directions on the high-dimensional surface, resulting in their projections onto two-dimensional planes appearing nearly as straight lines.

The ideal process parameters were identified based on a weight ratio of 0.4:0.4:0.2 for warpage, volume shrinkage, and clamping force. The outcomes of the simulation are presented in Table 5. The optimal process combination is as follows: melt temperature of 262.2 °C, mold temperature of 89.8 °C, filling time of 0.33 s, holding time of 1.37 s, and holding pressure of 60.28 MPa. The corresponding predicted warpage, volume shrinkage, and clamping force are 0.173 mm, 7.49%, and 15.83 tons, respectively. The simulation results for warpage and volume shrinkage showed deviations of less than 5% from the predicted values, while the deviation in clamping force was relatively large.

## 5. Conclusions

To achieve high-quality, cost-effective injection molding, this study focuses on new energy vehicle connector terminals, optimizing warpage, volume shrinkage, and clamping force as key objectives. The molding process parameters, including mold temperature, melt temperature, filling time, holding time, and holding pressure, are considered in the optimization. A combination of sampling strategies, numerical simulations, DBO-BP neural network modeling, and NSGA-II analysis is employed to explore the design space and simultaneously optimize the objectives. The research results from the input-output response analysis indicate that filling time significantly influences the quality characteristics of small, thin-walled parts, while holding pressure has a notable impact on the clamping force, with the magnitude of holding pressure largely determining the clamping force. Melt temperature and mold temperature are critical factors in controlling shrinkage. Using the DBO-BP model, an input-output response model is established, and multi-objective optimization of the nonlinear model is performed through weighted methods and NSGA-II. By establishing a predictive model, it becomes possible to rapidly evaluate quality indicators at any point within the parameter space, enabling global and continuous optimization of process parameters, which is a significant improvement over traditional design of experiments (DOEs) methods. Meanwhile, the application of a novel intelligent optimization algorithm in the modeling process further extends the applicability of such algorithms to complex engineering problems. The Pareto optimization results reveal a clear positive correlation between the volumetric shrinkage rate and the clamping force of the connector terminal—i.e., as shrinkage increases, the required clamping force also increases. In contrast, there is a negative correlation between shrinkage and warpage, reflecting the inherent trade-offs among multiple objectives. As shown in Table 5, the final optimization results indicate that the prediction errors for warpage and volumetric shrinkage are both within 5%, meeting the accuracy requirements for engineering applications. Although the error in clamping force prediction is relatively larger, it remains within an acceptable range. Further research is necessary to refine the model and improve prediction accuracy for the clamping force in future studies.

While the proposed methodology shows promising results, future research could focus on the following aspects:

1. Incorporating cooling channel design and its influence on thermal gradients and residual stress.
2. Real-time process control integration using sensor feedback and adaptive optimization.
3. Experimental validation of the predicted optimal parameters to confirm simulation accuracy.

## Figures and Tables

**Table 1:** Design variables and their ranges.

Design Variables	Lower Bound	Upper Bound
Melt temperature $T_{melt}$ (°C)	260	290
Mold temperature $T_{mold}$ (°C)	60	90
Injection time $t_{in}$ (s)	0.2	0.4
Holding pressure $P_{h}$ (MPa)	60	80
Holding time $t_{h}$ (s)	0.5	1.5

**Table 2:** Details of mesh division.

Contend	Detail
Type	Triangle
Number	49,966
Side length	0.4 mm
Aspect ratio	Maximum: 19.13, minimum: 1.16, and average: 1.61
Matching	Matching: 92.8% and mutual: 95.3%

**Table 3:** Material property of PA66GF20.

Material Data	Value
Melt/solid density (cm^3^)	1.34 g/1.16
Maximum shear stress (MPa)	0.5
Poisson’s ratio v12/v23	0.384/0.52
Shear modulus (MPa)	1947
Average modulus of elasticity (MPa)	5877.29
Tensile modulus (MPa)	7900
Ejection temperature (°C)	216
Recommended melt temperature (°C)	265–290
Recommended mold temperature (°C)	60–80

**Table 4:** Model evaluation indicators.

	MSE	RMSE	MAE	MAPE	$R^{2}$
DBOBP	0.177	0.421	0.220	3.21%	0.99628
BP	1.524	1.234	0.644	7.86%	0.96533

**Table 5:** Table of optimized results.

	$T_{melt}$ (°C)	$T_{mold}$ (°C)	$t_{in}$ (s)	$t_{h}$ (s)	$P_{h}$ (Mpa)	Warpage (mm)	Shrinkage (%)	Clamp (ton)
Pre	262.20	89.80	0.33	1.37	60.28	0.1729	7.491	15.83
Sim	262.20	89.80	0.33	1.37	60.28	0.1771	7.175	12.27
Error						2.3%	4.2%	22.5%

## Data Availability

The original contributions presented in the study are included in the article, further inquiries can be directed to the corresponding authors.
